# Analysis of Respiratory Muscle Strength Training in Amyotrophic Lateral Sclerosis (ALS) Patients: A Systematic Review

**DOI:** 10.7759/cureus.78903

**Published:** 2025-02-12

**Authors:** Ubaid Ansari, Jimmy Wen, Muhammad Karabala, Burhaan Syed, Ihab Abed, Daniel I Razick, Forshing Lui

**Affiliations:** 1 Neurology, California Northstate University College of Medicine, Elk Grove, USA; 2 Physical Medicine and Rehabilitation, California Northstate University College of Medicine, Elk Grove, USA; 3 Internal Medicine, California Northstate University College of Medicine, Elk Grove, USA; 4 Surgery, California Northstate University College of Medicine, Elk Grove, USA; 5 Clinical Sciences, California Northstate University College of Medicine, Elk Grove, USA

**Keywords:** amyotrophic lateral sclerosis, neurodegenerative disease, pulmonary rehabilitation, respiratory muscle strength training, respiratory muscle weakness

## Abstract

Respiratory muscle weakness is a significant contributor to morbidity and mortality in amyotrophic lateral sclerosis (ALS) patients. Respiratory muscle strength training (RMST) has emerged as a potential therapeutic approach to mitigate respiratory muscle weakness in ALS. Still, its efficacy and safety remain unclear due to conflicting evidence and methodological heterogeneity in existing studies. A systematic review was conducted across three databases (PubMed (United States National Library of Medicine, Bethesda, MD, USA), Embase (Elsevier, Amsterdam, Netherlands), and Cochrane Library (Cochrane, Alberta, Canada)) following Preferred Reporting Items for Systematic Reviews and Meta-Analyses (PRISMA) guidelines to assess the effectiveness of RMST in ALS patients. Eligible studies included comparative studies for RMST, focusing on outcomes such as maximum inspiratory pressure (MIP), maximum expiratory pressure (MEP), forced vital capacity (FVC), and ALS Functional Rating Scale (ALSFRS-R). Quality assessment was performed using the Cochrane Risk of Bias tool. This study included six studies, including 183 patients with a mean age of 58.0 years (49.6 to 63.2) and a mean follow-up time of 21.2 weeks (eight to 52). The average mean difference for ALSFRS-R (three studies), MIP (three studies), MEP (three studies), and FVC (two studies) were 2.062 (0.04 to 5.3), 2.285 (-8.145 to 10.8), 19.435 (10.86 to 21.7), and 7.23 (3.6 to 10.86), respectively. Complications related to RMST were poorly reported across studies. Secondary outcomes, such as depression scores, blood oxygen levels, and heart rate variability, showed promising trends but lacked consistency. Despite positive findings on respiratory muscle strength, RMST's efficacy in ALS management remains inconclusive. Challenges include methodological heterogeneity, limited sample sizes, and inadequate reporting of complications. Future research should focus on standardized protocols, larger sample sizes, longer follow-ups, and comprehensive assessment of adverse effects to clarify the role of RMST in ALS treatment.

## Introduction and background

Amyotrophic lateral sclerosis (ALS), also known as Lou Gehrig's disease, is a devastating neurodegenerative disorder characterized by the progressive degeneration of motor neurons in the brain and spinal cord, ultimately leading to muscle weakness, paralysis, and respiratory failure [1]. While the exact etiology of ALS remains elusive, its impact on respiratory function is profound, with respiratory muscle weakness being a significant contributor to morbidity and mortality in ALS patients [1]. Respiratory muscle weakness in ALS primarily affects the diaphragm and intercostal muscles, impairing the ability to breathe effectively and leading to respiratory insufficiency [2]. As the disease progresses, respiratory complications become increasingly prevalent, often becoming the primary cause of death in ALS patients. On average, individuals diagnosed with ALS succumb to the disease within three to five years, unless they opt for a tracheostomy, which may extend their lifespan by an average of two additional years [3]. However, up to 95% of patients with ALS in the United States decide against undergoing a tracheostomy [3]. Thus, interventions aimed at preserving or enhancing respiratory muscle strength are of paramount importance in the management of ALS. This will both potentially prolong survival as well as improve the quality of life for this patient population.

Respiratory muscle strength training (RMST) has emerged as a potential therapeutic approach to mitigate respiratory muscle weakness in ALS [4]. RMST involves targeted exercises designed to improve the strength and endurance of respiratory muscles, including the diaphragm, intercostals, and accessory muscles of respiration. RMST can be further subdivided into inspiratory muscle strength training (IMST) and expiratory muscle strength training (EMST). By enhancing respiratory muscle function, RMST holds the promise of preserving respiratory function and improving the quality of life for ALS patients [4]. Despite the growing interest in RMST as a therapeutic intervention for ALS, the evidence regarding its efficacy and safety remains inconclusive. While some studies have reported beneficial effects of RMST on respiratory muscle strength, pulmonary function, and respiratory symptoms in ALS patients, others have yielded conflicting results or failed to demonstrate significant improvements [5]. Moreover, the optimal timing, frequency, intensity, duration, and adherence of RMST in the context of ALS remain unclear, further complicating its clinical implementation. Given the heterogeneity of study designs, interventions, and outcomes in the existing literature, there is a critical need for a comprehensive synthesis of evidence to evaluate the actual effectiveness of RMST in ALS patients.

By synthesizing the data from randomized controlled trials, observational studies, and other relevant literature, this systematic review seeks to critically evaluate the potential benefits and limitations of RMST as a therapeutic intervention in ALS. Furthermore, this review aims to identify gaps in knowledge and areas for future research to help guide clinical practice and improve outcomes for ALS patients. We hypothesize that RMST will demonstrate significant improvements in respiratory muscle strength, pulmonary function, respiratory symptoms, and overall quality of life in ALS patients.

## Review

Methods

Search Strategy

A systematic search in accordance with the Preferred Reporting Items for Systematic Reviews and Meta-Analyses (PRISMA) a systematic search was conducted in PubMed (United States National Library of Medicine, Bethesda, MD, USA), Embase (Elsevier, Amsterdam, Netherlands), and Cochrane Library (Cochrane, Alberta, Canada) on September 24, 2024. All authors participated in the search, and the following keywords were used to identify articles that were suitable for this review: “amyotrophic lateral sclerosis”, “Respiratory muscle”, “strength training”, “expiratory”, “inspiratory”, and “forced vital capacity”. The article's date range was from 2009 to 2024. Ultimately, all included articles were in English, though this was not an exclusion criterion.

Article Selection

By the PICOT (Population, Intervention, Comparison, Outcome, Time) framework, eligibility and search strategies were established. The patient population included patients of all ages diagnosed with ALS. The intervention was RMST in this population. Comparative studies (randomized controlled trials) were included to compare muscle training effects with the control group. Outcomes included pre- and post-intervention maximum inspiratory pressure (MIP), maximum expiratory pressure (MEP), and forced vital capacity (FVC). Included studies consisted of randomized controlled trials and cohort studies.

The inclusion criteria focused on patients with confirmed diagnosed ALS and decreased respiratory function, measured by FVC. Exclusion criteria included patients who were not doing respiratory muscle exercise, case reports, reviews, and cadaveric reports. Title/abstract and full-text screening was done with a double-blinded dual-screening process on Covidence by two authors. If the decision to include the study was not unanimous, the disagreement was resolved with a third independent reviewer. All included studies underwent a thorough reference review to determine if there were additional studies to include. Finally, a manual search was performed by two authors to find additional studies that may have been missed in the original search of the four databases. This protocol is registered in the International Prospective Register of Systematic Reviews (PROSPERO) database as CRD42024540357.

Study Quality

The Cochrane Risk of Bias Tool and Methodological Index for Nonrandomized Studies (MINORS) criteria were used to determine the quality of the studies used in this review. Cochrane’s tool assesses domains such as sequence generation, allocation concealment, blinding of participants and personnel and blinding of outcome assessors, incomplete outcome data, selective outcome reporting, and other sources of bias, all graded on a “high” to “low” risk of bias scale. There was also an “unclear" option. MINORS criteria scores ranged from 0 (not reported), 1 (reported but inadequate), or 2 (adequately reported), with a maximum score of 16 for non-comparative studies and 24 for comparative studies. For this review, scores less than 8 were high risk of bias, 9-14 were intermediate risk of bias, and 15-16 were low risk of bias for non-comparative studies (cutoff for comparative studies were less than 14, 15-22, 23-24). Two authors went through each article using Cochrane and MINORS criteria individually and compared scores afterward. Any differences in the grading initiated a re-review of the article until a consensus was met.

Data Extraction/Analysis

Variables that were analyzed included title, author, publication date, study year, number of patients, mean age, mean follow-up time, BMI, preoperative and postoperative reported outcomes (Revised ALS Functional Rating Scale (ALSFRS-R) score, MIP and MEP, FVC), and complications. All extracted data were stored and analyzed via Google Sheets (Google Drive; Google, Mountain View, CA). Descriptive statistics (mean, percentage, standard deviations, ranges) were reported if applicable and available. Due to significant heterogeneity among the included studies, a meta-analysis was not performed, although originally planned.

Results

Article Selection

**Figure 1:**
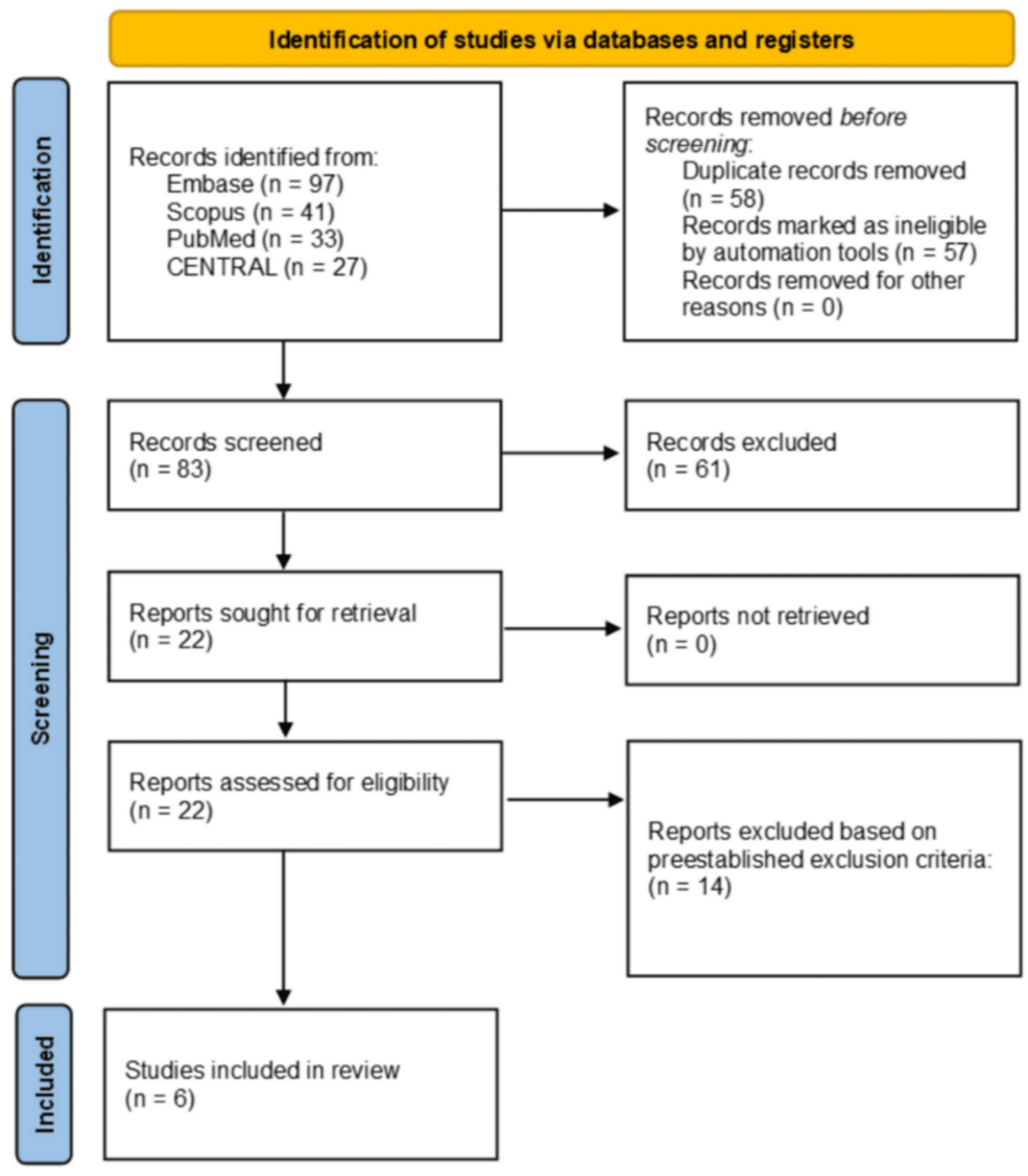
Preferred Reporting Items for Systematic Reviews and Meta-Analyses (PRISMA) diagram depicting article selection process

The initial search yielded 198 records for screening. After duplicate records were removed (n=58) and records were marked as ineligible by automation tools (n=57), a total of 83 texts remained. Upon completion of title and abstract screening (n=83), a total of 22 texts remained. A full-text review led to the exclusion of 14 articles due to either being not performance-focused (six) or the wrong study design (two), leaving six total studies included in the systematic review (Figure 1) [6-11].

Patient Characteristics of Included Studies

There were six total studies used in this systematic review. A total of 183 patients diagnosed with ALS were included, of which 109 were male and 74 were female. The mean age of the patients included was 58.0 years (49.6 to 63.2). The mean follow-up time for patients included in this study was 21.2 weeks (eight to 52). All 10 studies had a level of evidence (LOE) of II. Table 1 summarizes the study characteristics and patient demographics included in this study.

**Table 1:** Patient characteristics LOE, level of evidence

Author	Journal	Study year(s)	LOE	Number of patients (M/F)	Mean age (years)	Mean follow-up (weeks)
Cheah et al., 2009 [6]	Amyotrophic Lateral Sclerosis and Frontotemporal Degeneration	2007-2008	2	19 (12/7)	54.4	12
Pinto et al., 2012 [7]	Amyotrophic Lateral Sclerosis and Frontotemporal Degeneration	2011	2	26 (18/8)	57	32
Plowman et al., 2016 [8]	Muscle Nerve	-	2	25 (14/11)	62.2	15
Plowman et al., 2019 [9]	Muscle Nerve	-	2	48 (29/19)	61.6	8
Vicente-Campos et al., 2022 [10]	Journal of Clinical Medicine	2021	2	20 (9/11)	49.6	8
Plowman et al., 2023 [11]	Neurology	-	2	45 (27/18)	63.2	52

The main outcomes reported in most of these studies were the ALS Functional Rating Scale (ALSFRS), MIP, MEP, and FVC. In general, the four studies that were analyzed using the Cochrane risk of bias tool displayed a lower risk of bias for six out of seven domains. For the first domain, there was an unclear risk of bias for the sequence not being sufficiently randomly generated for one study [7]. As for the second domain, there was an unclear risk of bias regarding the allocation of the drug and placebo assignments for two studies [7,9]. For the third domain, there was a high risk of bias in terms of blinding of participants and personnel in one study and an unclear risk of bias for this in another study [7,9]. There was also an unclear risk of bias for blinding of outcome assessors in one study, due to the lack of mention of any measures used to confirm blinding [7]. It was determined that there was a low risk of bias for domains three, four, and five for all studies. These findings are summarized in Table 2.

**Table 2:** Cochrane risk of bias

Author	Sequence generation	Allocation concealment	Blinding of participants and personnel	Blinding of outcome assessors	Incomplete outcome data	Selective outcome reporting	Other source of bias
Cheah et al., 2009 [6]	Low	Low	Low	Low	Low	Low	High
Pinto et al., 2012 [7]	Unclear	Unclear	High	Unclear	Low	Low	High
Plowman et al., 2019 [8]	Low	Unclear	Unclear	Low	Low	Low	High
Plowman et al., 2023 [11]	Low	Low	Low	Low	Low	Low	High

Out of six studies, five studies were comparative, and one was non-comparative, with an average MINORS score of 23.4 and 14, respectively. Thus, the risk of bias was determined to be low in four studies and intermediate in two studies. MINORS scores for all studies can be found in Table 3.

**Table 3:** Methodological quality and risk of bias 2, Reported Adequately; 1, Reported but not adequately; 0, not reported

Author	Clearly stated aim	Inclusion of consecutive patients	Prospective data collection	Endpoints appropriate to study aim	Unbiased assessment of study endpoint	Follow-up period appropriate to study aim	Loss to follow-up less than 5%	Prospective calculation of study size	Adequate control group	Contemporary groups	Baseline equivalence of groups	Adequate statistical analyses	Total score
Cheah et al., 2009 [6]	2	2	2	2	2	2	2	2	2	2	2	2	24
Pinto et al., 2012 [7]	2	2	2	2	2	2	2	2	2	2	2	2	24
Plowman et al., 2016 [8]	2	2	2	2	2	2	1	1	0	0	0	0	14
Plowman et al., 2019 [9]	2	2	2	2	2	2	2	1	2	2	2	2	23
Vicente- Campos et al., 2022 [10]	2	2	2	2	1	2	2	1	2	2	2	2	22
Plowman et al., 2023 [11]	2	2	2	2	2	2	2	2	2	2	2	2	24

Revised Amyotrophic Lateral Sclerosis Functional Rating Scale

Four of the studies that were analyzed compared the mean difference of ALSFRS-R scores to control groups that had not undergone RMST. Vicente-Campos et al. were the only ones to have found a significant mean difference of 5.3 points (p=0.043) [10]. Cheah et al. reported a mean difference of 0.04 ± 0.73 points (p=0.95) [6]. Pinto et al. had a mean difference of 0.846 ± 1.455 points (p=0.566) [7]. While Plowman et al. did not report specific numbers, they mentioned that the value was insignificant with a p=0.84 [9]. The average between the three studies that reported a mean difference of ALSFRS-R is 2.062 points (0.04 to 5.3). These findings are summarized in Table 4.

**Table 4:** Amyotrophic lateral sclerosis (ALS) functional outcome variables ALSFRS-R, Revised Amyotrophic Lateral Sclerosis Functional Rating Scale; NR, not reported

		ALSFRS-R difference from baseline (p-value)		Maximum inspiratory pressure difference from baseline (% of predicted) (p-value)		Maximum expiratory pressure difference from baseline (% of predicted) (p-value)	Forced vital capacity difference from baseline (p-value)		Complications
										Cheah et al., 2009 [6]	Mean difference	0.04 ± 0.73 points (p= 0.95)		6.10 ± 6.93% (p=0.39)		1.13 ± 3.33% (p=0.74)	4.59 ± 3.02% (p=0.15)		1 patient in the experimental group was lost to follow-up due to death
Pinto et al., 2012 [7]	Mean difference	0.846 ± 1.455 points (p=0.566)		-8.154 ± 10.51 cm H2O (p=0.445)		-7.615 ± 11.84 cm H2O (p=0.526)	10.86 ± 7.324% (p=0.151)		1 patient in Group 1 was lost due to rapidly progressive spasticity. 1 patient in Group 2 was lost to follow-up										
										Plowman et al., 2016 [8]	Mean difference	NR		NR		17.17 ± 5.87 cm H2O (p=0.01)	NR		10 patients dropped out
Plowman et al., 2019 [9]	Mean difference	NR (p=0.84)		NR		NR (p=0.009)	NR (p=0.86)		1 dropped out from each group. One for cancer diagnosis requiring chemotherapy. Another no longer wished to participate.										
										Vicente-Campos et al., 2022 [10]	Mean difference	5.3 points (p=0.043)		10.8 ± 7.4 cm H2O (p<0.001)		NR	NR		All 20 completed
Plowman et al., 2023 [11]	Mean difference	NR		4.2 ± 8.9 cm H2O (p=0.33)		21.7 ± 15.3 cm H2O (p=0.004)	3.6 ± 12.1% (p=0.60)		NR										

Maximum Inspiratory Pressure

Of the four studies that measured MIP, only Vicente-Campos et al. were found to have a statistically significant increase in MIP after RMST as compared to the control, with a mean difference measuring 10.8 ± 7.4 cm H_2_O (p<0.001) [10]. Pinto et al. and Plowman et al. both reported an insignificant mean difference of -8.145 ± 10.51 cm H_2_O (p=0.445) and 4.2 ± 8.9 cm H_2_O (p=0.33), respectively [7,11]. Cheah et al. reported a 6.10 ± 6.93% (p=0.39) predicted value for the MIP difference between the experiment and control group [6]. The average mean difference of MIP between both measured groups was 2.285 cm H_2_O (-8.145 to 10.8). These findings are summarized in Table 2.

Maximum Expiratory Pressure

MEP was measured in five of the trials that were analyzed. Three trials reported a significant mean difference in MEP between the experiment and control, measuring at 17.17 ± 5.87 cm H_2_O (p=0.01) and 21.7 ± 15.3 cm H_2_O (p=0.004) for Plowman et al. [8] and Plowman et al. [11], respectively. Although no specific numbers were reported, Plowman et al. also found a significant difference with p=0.009 [9]. Pinto et al. found the mean difference to be 10.86 ± 7.324 cm H_2_O (p=0.151), and Cheah et al. found MEP to measure at 4.59 ± 3.02% (p=0.15) of predicted value between the experiment and control [6,7]. MEP had the largest average mean difference between the primary outcomes with a measurement of 19.435 cm H_2_O (10.86 to 21.7). These findings are summarized in Table 2.

Forced Vital Capacity

FVC was reported in four of the studies included in the review. None of the papers had reported a clinically significant difference in pre- and post-intervention of RMST when compared to control. Pinto et al. and Plowman et al. reported a mean difference of FVC of 10.86 ± 7.324% predicted value (p=0.151) and 3.6 ± 12.1% predicted normal value (p=0.60), respectively [7,11]. Cheah et al. found that there was a 4.59 ± 3.02% (p=0.15) predicted value for FVC difference between the experiment and control group [6]. Although Plowman et al. did not report specific numbers for FVC, they found there was no statistical significance between both groups [9]. Of the two studies that reported a mean difference for FVC, the average between them was 7.23% of the predicted value (3.6 to 10.86). These findings are summarized in Table 2.

Secondary Outcomes

A few secondary measures that were statistically significant between the RMST experimental group and control were noted between four different articles and subjected to review. Pinto et al. measured depression based on the Hamilton Depression Rating Scale (HRSD) and found that the RMST group had significantly lower scales of depression, with a mean difference between both groups at 1.769 ± 0.848 points (p=0.05). They have also found significantly higher blood oxygen SpO_2_ levels in the experimental group with a mean difference of 0.915 ± .435 SpO2 (p=0.046) [7].

Plowman et al. included a swallow Dynamic Imaging Grade of Swallowing Toxicity (DIGEST) score that measures the pharyngeal swallow functions that are primarily used for measuring dysphagia using liquids with different consistencies [12]. They found that there was no change in the DIGEST score in the patients within the active group (4.3% change). However, the control group scores had worsened from pre- and post-intervention periods with an 18.1% increase in DIGEST score, indicating worsening dysphagia (p=0.02) [9].

Vicente-Campos et al. measured a wide number of baseline characteristics and found that the experimental group had statistically significant lower measurements of heart rate and R-wave to R-wave interval with a mean difference of −8.80 ± 11.47 bpm (p=0.023) and 78.30 ± 75.41 ms (p=0.019) [10].

Complications

Vicente-Campos et al. and Plowman et al. had no complications related to their study [10,11]. All patients that were recruited had successfully completed their assignments. In Cheah et al., one patient in the experimental group was lost to follow-up due to death [6]. Pinto et al. had two total patients that were lost due to follow-up, one in each of the groups [7]. Plowman et al. had six participants who withdrew following the baseline evaluation, and during treatment, four more individuals withdrew [8]. Plowman et al. also had a patient from each group who was lost to follow-up, one who no longer wished to participate, and another who received a diagnosis of cancer [9]. These findings are summarized in Table 2.

Discussion

This systematic review encompassed six studies, with a total of 183 ALS patients, to assess the outcomes of RMST in this patient cohort. The main findings were that RMST provided statistically significant or numerical non-significant increases in MIP, MEP, and ALSFRS-R, but not FVC with low rates of complications. However, not all of the studies reported all of these outcomes or complications and thus these findings must be carefully considered.

Functional Outcomes

Respiratory muscle weakness is a major cause of morbidity and mortality in ALS patients as respiratory issues develop quickly regardless of the site of disease onset (extremity or bulbar) [13] The diaphragm is the most important contributor to ventilation and can be strengthened via respiratory training similar to the mechanisms of skeletal muscle training. However, there has been controversy regarding respiratory training as critics have discussed the deleterious effects on motor neurons and hastened progression of the disease course [13]. FVC has been proposed as an important early clinical assessment as it has been discovered that patients can present with a decreased FVC and respiratory muscle strength even with asymptomatic breathing function (normal lung volumes) [14].

RMST appears to have a positive effect on respiratory muscle strength in ALS patients as indicated in the included studies. These findings are supported by respiratory muscle interventions in other neurological conditions such as stroke, Parkinson’s, and neuromuscular disease [15]. The length of intervention may also affect the outcomes assessed as longer-term follow-ups may not confer a statistically significant benefit compared to shorter follow-ups due to the rapidly progressive nature of ALS. However, in Pinto et al.’s survival analysis up to 32 months, they found that inspiratory muscle training (IMT) and subsequent improved phrenic nerve response were significant independent prognostic factors for ALS patients. Additionally, IMT patients had a significantly higher survival rate albeit compared to a historical control ALS group (p<0.001) [16]. A recent meta-analysis of RMST demonstrated an improvement in MIP and MEP albeit not in a significant manner [15]. Vicente-Campos et al. utilized a novel POWERbreathe IMT device (POWERbreathe International Ltd., UK) that led to significant improvement in MIP and an interesting finding of a lowered resting heart rate and increased heart rate variability [10,17]. Autonomic nervous system dysfunction can occur at any stage of ALS and ALS patients are associated with high resting heart rates and lowered heart rate variability. Decreased heart rate variability is also associated with patients with lower lung capacity and an increased need for ventilatory support [18]. This finding by Vicente-Campos et al. is not well characterized and future studies are required to assess the clinical correlation of IMT on autonomic functions.

The majority of the preliminary studies focused on the effect of either inspiratory training or expiratory training. Plowman et al. built upon these studies and aimed to examine if there is a synergistic effect of training both inspiratory and expiratory functions. They found a statistically significant improvement in MEP, cough function, and one-year ALSFRS-R bulbar decline rates [11]. Although this supports the positive effect of expiratory training on MEP, there was not a significant improvement in MIP [8,9]. However, the authors attributed the decreased overall MEP and MIP values to their study design as they reduced the overall resistance loads to avoid overtraining, given that the training load will be doubled with both inspiratory and expiratory exercises [11]. Several IMT studies have included progressive loads of up to 60% which provides a sufficient stimulus to induce neuromuscular adaptations and confers a survival benefit [6,11,16]. Therefore, future studies can incorporate an appropriately increased load to observe any additional survival benefits of this protocol.

MIP has been used in several of the included studies as it is a quick and non-invasive measurement for inspiratory muscle strength. The reliability of this measurement has been called into question as it is highly dependent on the level of effort exerted which can be affected by buccal weakness with ALS patients and the mouthpiece used (flanged, cardboard, rubber, etc.), preventing a standardized measurement [13]. Sniff nasal inspiratory pressure (SNIP) is a more reliable measure for inspiratory muscle strength with a sensitivity of 97% and is easier to perform in ALS patients even for those with perioral muscle weakness [19,20]. MEP is another simple and non-invasive method of measuring early indications of respiratory muscle weakness. However, contrary to MIP and SNIP, MEP does not seem to have a significant association with respiratory symptoms most notably with FVC and FEV1 (restrictive patterns) [21]. Normal MIP and MEP values are still significantly associated with ALS survival [21]. The ALSFRS-R questionnaire is another common tool for measuring disease progression in ALS patients and is correlated with other quality-of-life questionnaires [13]. SNIP is also significantly correlated with the ALSFRS-R score as well [21]. Another significant correlation is between ALSFRS-R score and slowed vital capacity (SVC) for disease progression. ALS patients with smaller declines in SVC over time are more likely to have smaller decreases in the respiratory subdomain of ALSFRS-R [22]. SVC also is related to changes in dyspnea, respiratory insufficiency, and the total ALSFRS-R score [22]. However, the validation for the respiratory subscore of the ALSFRS-R questionnaire has been called into question as no correlation was found between the subscore and FVC [23]. Pinto et al. mentioned that respiratory decline is not entirely based on respiratory muscle failure and several other factors such as deconditioning and fatigue must be appreciated [23]. Therefore, future studies should also aim to find validated outcomes to evaluate their patient cohorts to better place findings within the context of ALS progression.

RMST does not appear to have a significant effect on FVC values in ALS patients. This may be in part explained by the principle of specificity of neuroplasticity and skeletal muscle adaptations [24,25]. Respiratory muscles are functionally skeletal muscles and respond similarly when trained [5]. Therefore, as RMST would induce changes in pressure generation indicated by improvements in MIP and MEP, lung volume recruitment such as breath stacking would theoretically increase FVC and total lung capacity [11]. Thus, future training programs can analyze the effect of both RMST and lung volume recruitment in ALS patients. However, meta-analysis shows the beneficial effects of RMST on other neurological and neurodegenerative diseases (e.g., multiple sclerosis, Parkinson’s, spinal cord injury, stroke) on IMT, EMT, and FVC [5].

Complications

Across the studies, complications were not reported adequately or not reported at all. Thus, it remains uncertain if RMST causes adverse effects, and future studies must pay close attention to potential adverse effects that can arise during the interventions.

Future Indications

ALS patient recruitment and retention remains a challenge for researchers such as obtaining a sufficient sample size and power, patients that fit the study inclusion criteria (disease duration, FVP, etc.), and eligibility requirements (age, sex) [26]. The development of the National ALS Registry’s research notification mechanism (RNM) has aided researchers in recruiting patients for clinical trials. However, despite this tool, it is estimated that 60% of ALS patients are ineligible for clinical trials [27]. This raises the question of how generalizable the findings are in ALS trials as a substantial number of ALS subgroups are excluded or underrepresented with strict exclusion criteria. Despite these limitations, the results provide evidence to support the hypothesis that respiratory muscle training can favorably affect the respiratory muscles of ALS patients. There also appears to be a load-dependent relationship with training outcomes, but it must be implemented appropriately to decrease the risk of unintentional complications.

Future studies should include, if possible, larger sample sizes and longer training protocols in a randomized manner to minimize bias. This will allow better determination of the most optimal training intensity and protocols in this patient population. Additionally, comparisons between the bulbar and spinal onset of ALS can better elucidate the effects of these symptoms on respiratory training outcomes.

Limitations

Several limitations must be addressed with this systematic review. First, there was considerable heterogeneity in training protocol and outcomes measured, thus preventing a definitive conclusion on the outcomes of respiratory training. Second, there were a limited number of available trials, and a small number of patients included. Of the available patients, they generally were early on in their disease process and these findings cannot be extrapolated toward ALS patients across the continuum. There is a current hypothesis that early intervention via these RMSTs can reduce the rate of decline, but future studies are needed to confirm the efficacy of RMST programs for individuals at later stages of ALS. Third, complications were rarely if at all reported in these studies and future studies are needed to determine the risk ratio of RMST. Fourth, only English language studies were included.

## Conclusions

Despite the profound impact of respiratory muscle weakness on ALS progression and outcomes, the efficacy of RMST remains an area of debate. This review highlights the positive effects of RMST on respiratory muscle strength, as evidenced by improvements in maximal inspiratory and expiratory pressures in ALS patients. These findings are consistent with the broader literature on respiratory muscle interventions in neurological conditions, suggesting a potential avenue for improving respiratory function in ALS. However, challenges and controversies persist, particularly regarding the optimal duration and intensity of RMST, as well as concerns regarding potential adverse effects on disease progression. Additionally, while some studies demonstrate favorable outcomes with RMST, others report conflicting results or fail to show significant improvements in functional outcomes such as FVC. The heterogeneity in study designs, patient populations, and outcome measures further complicates the interpretation of findings. Future research directions should focus on addressing these limitations by employing larger sample sizes, longer-term follow-ups, and standardized training protocols. Furthermore, investigations into the differential effects of RMST on bulbar and spinal onset ALS subtypes could provide valuable insights into tailored therapeutic approaches. Additionally, efforts to enhance ALS patient recruitment and retention in clinical trials are imperative to ensure the generalizability and applicability of findings across diverse patient populations. In summary, while RMST holds promise as a therapeutic intervention for respiratory muscle weakness in ALS, further research is needed to elucidate its optimal implementation and long-term effects on disease progression and patient outcomes. By addressing these gaps in knowledge, we can advance our understanding of RMST in ALS management and incorporate multidisciplinary approaches that consider individual patient characteristics and disease trajectories. This will ultimately improve the quality of life and survival outcomes for individuals living with this devastating disease.
